# Antibacterial and Cytotoxic Activity of Compounds Isolated from* Flourensia oolepis*

**DOI:** 10.1155/2015/912484

**Published:** 2015-12-27

**Authors:** Mariana Belén Joray, Lucas Daniel Trucco, María Laura González, Georgina Natalia Díaz Napal, Sara María Palacios, José Luis Bocco, María Cecilia Carpinella

**Affiliations:** ^1^Fine Chemicals and Natural Products Laboratory, School of Chemistry, Catholic University of Córdoba, Avda Armada Argentina 3555, X5016DHK Córdoba, Argentina; ^2^CIBICI CONICET and Department of Clinical Biochemistry, Faculty of Chemical Science, National University of Córdoba, Haya de la Torre and Medina Allende, Córdoba, Argentina

## Abstract

The antibacterial and cytotoxic effects of metabolites isolated from an antibacterial extract of* Flourensia oolepis* were evaluated. Bioguided fractionation led to five flavonoids, identified as 2′,4′-dihydroxychalcone (**1**), isoliquiritigenin (**2**), pinocembrin (**3**), 7-hydroxyflavanone (**4**), and 7,4′-dihydroxy-3′-methoxyflavanone (**5**). Compound** 1** showed the highest antibacterial effect, with minimum inhibitory concentration (MIC) values ranging from 31 to 62 and 62 to 250 *μ*g/mL, against Gram-positive and Gram-negative bacteria, respectively. On further assays, the cytotoxic effect of compounds** 1**–**5** was determined by MTT assay on acute lymphoblastic leukemia (ALL) and chronic myeloid leukemia (CML) cell lines including their multidrug resistant (MDR) phenotypes. Compound** 1** induced a remarkable cytotoxic activity toward ALL cells (IC_50_ = 6.6–9.9 *μ*M) and a lower effect against CML cells (IC_50_ = 27.5–30.0 *μ*M). Flow cytometry was used to analyze cell cycle distribution and cell death by PI-labeled cells and by Annexin V/PI staining, respectively. Upon treatment,** 1** induced cell cycle arrest in the G_2_/M phase accompanied by a strong induction of apoptosis. These results describe for the first time the antibacterial metabolites of* F. oolepis* extract, with** 1 **being the most effective. This chalcone also emerges as a selective cytotoxic agent against sensitive and resistant leukemic cells, highlighting its potential as a lead compound.

## 1. Introduction

Since the discovery of chemotherapy, scientists have developed a vast arsenal of bioactive agents enabling the successful treatment of a huge variety of diseases, including bacterial infections and cancer. However, over time, the treatment of these ailments is frequently associated with side effects and the development of resistance, rendering the drugs useless [1, 2]. This has increased the importance of the search for new entities with antibacterial and anticancer properties.

The role of natural products in drug discovery remains critical. Sixty-five percent of the 118 new chemical entities (NCE) approved between 1981 and 2010 for indication as antibacterial drugs and 34% of the 128 approved as anticancer drugs corresponded to natural products and semisynthetic derivatives obtained from these natural precursors [3]. The fact that natural products have 40% more chemical scaffolds than synthetic chemistry means that they have a considerable advantage in continuing to provide new commercial drug leads [4]. The key is to efficiently and effectively access this diversity [4]. Among sources of natural active compounds, plants are a promising starting point [5]. Approximately one-third of the top-selling drugs have been derived from plant metabolites [6] and there are 190 examples of clinical trials involving pure or combined plant compounds for treating different ailments currently reported (https://www.clinicaltrials.gov/, US Institute of Health, query “plant drugs”; only herbal extracts or plant-derived pure compounds administered as therapeutic agents were considered). The plant world is still far from being totally explored, however, particularly the native flora from Argentina [7].* Flourensia oolepis *S. F. Blake (Asteraceae), commonly known as “chilca,” is a woody bush widely distributed in the high areas of the provinces of Córdoba and San Luis, in central Argentina [8]. The antibacterial activity of the ethanol extract obtained from this plant was previously reported by our group [7]. The current study aims to gain insight into the metabolites responsible for this effect.

The cytotoxic activity of the compounds isolated against leukemic cell lines and their multidrug resistant (MDR) counterparts was also investigated, and this was extended to determine the mechanism involved in leukemic cell toxicity.

To our knowledge, this is the first detailed study about the constituents of* F. oolepis* that show these pharmacological properties.

## 2. Materials and Methods

### 2.1. Plant Material and Extract Preparation

Aerial parts of the native plant* F. oolepis* S. F. Blake were collected from the hills of Córdoba Province, Argentina, in December 2005. A voucher specimen was deposited in the “Marcelino Sayago” Herbarium of the School of Agricultural Science, Catholic University of Córdoba (UCCOR 23). The authorization for the use of the plant is available from the authors. Crushed air-dried material (200 g) was extracted by 48 h maceration with 700 mL of ethanol, and the yield of the extract, obtained after solvent removal and expressed as percentage weight of air-dried crushed plant material, was 23 g%.

### 2.2. Chemicals, Equipment, and Reagents

3-(4,5-dimethylthiazol-2-yl)-2,5-diphenyltetrazolium bromide (MTT) was purchased from Sigma-Aldrich CO (St Louis, MO, USA). Doxorubicin hydrochloride 99.8% (DOX, Synbias Pharma Ltd.) was purchased from Nanox Release Technology (Buenos Aires, Argentina).

Gentamicin sulfate (potency: 550–590 *μ*g/mg) and erythromycin (potency: 863 *μ*g/mg) were provided by Laboratorio Fabra S.A, Buenos Aires, Argentina, and Unifarma, Buenos Aires, Argentina, respectively.

^1^H- and ^13^C-NMR and two-dimensional spectra were recorded with a Bruker AVANCE II 400 spectrometer (Bruker Corporation, Ettlingen, Germany) with tetramethylsilane (TMS) as the internal reference. HPLC was performed on a Shimadzu LC-10 AS (Shimadzu Corp., Tokyo, Japan), equipped with a Phenomenex Prodigy 5 *μ* ODS (4.6 mm i.d. × 250 mm) reversed-phase column. The mobile phase was water/methanol/trifluoroacetic acid 65 : 35 : 1 with detection at 365 nm.

Flow cytometry analysis was performed in a Becton-Dickinson (BD) FACS Canto II flow cytometer (BD Biosciences, USA).

### 2.3. Bioguided Isolation of the Active Principles from* Flourensia oolepis*

The antibacterial ethanol extract of* F. oolepis *(12 g) was initially subjected to vacuum liquid chromatography on silica gel (622 g, 63–200 *μ*m, 11.0 × 24.0 cm; Macherey & Nagel) eluted with a step gradient of hexane/diethyl ether (Et_2_O)/methanol (MeOH) to yield 26 fractions, which were combined in 6 groups according to their thin layer chromatography (TLC) profile (F1 to F6). Of these, fraction F3 eluted with hexane/Et_2_O 50 : 50 and F5, eluted with 100% Et_2_O, demonstrated bactericidal activity at 2 mg/mL and were therefore submitted to additional separation methods for further purification. F3 was processed by radial preparative chromatography using an isocratic mobile phase of hexane/Et_2_O 70 : 30. The fractions obtained were combined in 5 groups in accordance with the TLC analysis (F_3_1 to F_3_5). From fraction F_3_4, compound** 3** was obtained by spontaneous crystallization (97.8% purity, by HPLC). The pure compound, the remaining F_3_4 containing traces of** 3** and another substance, as well as the rest of the fractions, were tested for antibacterial activity. Only** 3** and the remaining F_3_4 exerted bactericidal effects at 2 mg/mL. The latter was further fractionated by preparative TLC (Analtech DE, USA; mobile phase: toluene/ethyl acetate/glacial acetic acid 8 : 2.5 : 0.4) to finally obtain compound** 1** (99.9% purity, by HPLC).

Fraction F5 was subjected to column chromatography (130 g, 35–70 *μ*m, 3.0 × 60 cm; Fluka) using 100% Et_2_O as the mobile phase. The resulting fractions were placed in 14 groups according to TLC monitoring (F_5_1 to F_5_14). Of these, F_5_2 showed bactericidal activity and was further fractionated by column chromatography on silica gel (60 g, 35–70 *μ*m, 3.0 × 60 cm; Fluka) performed with hexane/Et_2_O/glacial acetic acid 8 : 2 : 0.4. The fractions obtained were combined in 7 groups according to their TLC profile (F_5.2_1–F_5.2_7). Their antibacterial activity was evaluated at 2 mg/mL, and F_5.2_2 and F_5.2_5 were seen to be active. Both groups were separately submitted to solid phase extraction (Sep-Pak plus C18, Waters, Ireland) using water/MEOH/trifluoroacetic acid 65 : 35 : 1 as the mobile phase, to afford compound** 2** (99.1% purity, by HPLC) from F_5.2_2 and compounds** 4** (96.4% purity, by HPLC) and** 5** (97.2% purity, by HPLC) from F_5.2_5.

Compounds** 1**–**5** were identified by ^1^H NMR and ^13^C NMR, comparing their spectra (copies of the original spectra are obtainable from the corresponding author) with reference data from literature, as 2′,4′-dihydroxychalcone C_15_H_12_O_3_ (**1**;* m/z* 240.2) [9, 10], isoliquiritigenin C_15_H_12_O_4_ (**2**;* m/z* 256.2) [11], pinocembrin C_15_H_12_O_4_ (**3**;* m/z* 256.2) [8], 7-hydroxyflavanone C_15_H_12_O_3_ (**4**;* m/z* 240.2) [12], and 7,4′-dihydroxy-3′-methoxyflavanone C_16_H_14_O_5_ (**5**;* m/z* 286.3) [13]. The compounds were quantified by HPLC and their yields in g per 100 g of dried and crushed plant material were 1.24, 0.06, 1.14, 0.3, and 0.06 for** 1**–**5**, respectively.

### 2.4. Microorganisms and Preparation of Inocula

The antibacterial activity assays were carried out on strains of* Enterococcus faecalis* (Andrews and Horder) Scheifer and Klipper-Balz (ATCC 29212)*, Escherichia coli* (Migula) Castellani and Chalmers (ATCC 25922)*, Pseudomonas aeruginosa* (Schroeter) Migula (ATCC 27853), and* Staphylococcus aureus* subsp.* aureus* Rosenbach (ATCC 6538) and on a clinical isolate of tetracycline, erythromycin, and polymyxin-resistant* Proteus mirabilis* (Pr2921) [14]. This isolate was identified by conventional biochemical assays and by the commercial system API20E (bioMérieux SA, Mercy L'Etoile, France). Antimicrobial resistances were determined by disk diffusion susceptibility testing (Kirby-Bauer Method) according to CLSI [15]. Bacterial suspensions were prepared on sterile saline from each organism grown overnight. Turbidity was spectrophotometrically adjusted to the McFarland 0.5 standard. Dilutions were carried out with sterile saline to give an adjusted concentration of 1.5 × 10^7^ colony-forming units/mL.

### 2.5. Antibacterial Activity Test and Determination of MICs and MBCs

MICs were determined by the agar dilution test according to CLSI [15] with some modifications [7]. Briefly, the plate count agar medium (PCA, Oxoid Ltd, UK) was added in duplicate to the suitable amount of fraction or pure compound previously dissolved in ethanol. The final concentration of solvent was 2% (no adverse effects were observed at this concentration). After solidification of the agar in 6-well plates (Greiner Bio-One, Germany), 2 *μ*L of each bacterial suspension was placed on the agar surface. Three independent experiments were carried out. Plates were incubated in ambient air at 37°C for 24 h. For reading, visual observations were carried out. Nitro blue tetrazolium (NBT, Sigma-Aldrich CO, USA) solution in saline phosphate buffer (PBS) was applied on one spot for confirmation. Plates containing only the culture medium, with or without the addition of the dissolution solvent, were used as controls. Positive controls with commercial gentamicin sulfate or erythromycin dissolved in sterile water or ethanol, respectively, were simultaneously carried out. The minimum inhibitory concentration (MIC) was defined as the lowest concentration that completely inhibits growth of the microorganism.

The bactericidal effect was further studied from each concentration of product showing growth inhibition. For this study, 0.5 mm portions of agar, coincident with the place at which inocula had been placed and showing negative growth at 24 hr (without NBT), were cut and transferred to brain-heart infusion broth (BHI, Oxoid Ltd, UK). Tubes were incubated in ambient air at 37°C for five days. At the end of this period, the minimum bactericidal concentration (MBC) values, defined as the lowest concentration with absence of turbidity, were recorded.

### 2.6. Cell Lines and Culture Conditions

CCRF-CEM acute lymphoblastic leukemia (ALL) [16] and K562 chronic myeloid leukemia (CML) [17] cells and their MDR P-glycoprotein overexpressing variants [18, 19], CEM/ADR5000 and Lucena 1, respectively, were a generous gift from Dr. T. Efferth (Institute of Pharmacy and Biochemistry, Johannes Gutenberg University, Mainz, Germany) and Dr. V. Rumjanek (Instituto de Bioquímica Médica Leopoldo de Meis, Universidade Federal do Rio de Janeiro, Rio de Janeiro, Brazil), respectively. The HEK293T non-tumor derived cell line [20] and human isolated mononuclear cells (PBMC) were also tested. Leukemic and PBMC cells were grown in Roswell Park Memorial Institute (RPMI) 1640 medium (Invitrogen Life Technologies, CA, USA) while the HEK293T cell line was grown in Dulbecco's Modified Eagle's medium (DMEM, Invitrogen Life Technologies, CA, USA), both of these media supplemented with 10% heat-inactivated fetal bovine serum (FBS, PAA Laboratories, Pasching, Austria), 2 mM glutamine (Invitrogen Life Technologies, CA, USA), and penicillin (100 units/mL) streptomycin (100 *μ*g/mL) (Invitrogen Life Technologies, CA, USA). Cells were cultured at 37°C in a 5% CO_2_ humidified environment.

CEM/ADR5000 cells were exposed once a week to gradually increasing doses of DOX from 1.7 to 8.6 *μ*M. The latter concentration was then used for maintenance of the cells. Lucena 1 were continuously cultured in the presence of 60 nM DOX. Both cell lines were grown in drug-free medium 3-4 days before the experiments. Overexpression of P-glycoprotein (P-gp) in both MDR variants was verified by flow cytometry using FITC-labeled mouse anti-human P-gp (BD Pharmingen, USA). Cells were subcultured twice a week from frozen stocks and used before the 20th passage. All experiments were performed with cells in the logarithmic growth phase with cell viabilities over 90%, determined by trypan blue staining.

### 2.7. Isolation of Human Mononuclear Cells from Peripheral Blood

Peripheral blood mononuclear cells (PBMC) were collected from fresh heparinized blood and separated by density gradient centrifugation (Ficoll) as described by Rennó et al. [21]. As the current study required samples from healthy human volunteer donors, ethical approval was provided by the Catholic University of Córdoba Research Ethics Board. Written signed consents were obtained from donors.

### 2.8. Cell Proliferation Assay

To investigate the cytotoxic potential of the isolated compounds, the MTT colorimetric assay was performed [22]. Briefly, 5 × 10^4^ cells suspended in 100 *μ*L of growth medium were seeded in 96-well plates (Greiner Bio-One, Germany) containing 100 *μ*L of medium in the presence of serial twofold dilutions of each tested compound previously dissolved in ethanol (1% v/v, no adverse effects on cell growth were observed at this concentration). The compounds were evaluated at a final maximum concentration of 100 *μ*M. After 72 hours, 20 *μ*L of 5 mg/mL solution of MTT in sterile PBS was added to each well and further incubated for 4 h. Then, the supernatants were removed and replaced with 100 *μ*L DMSO to solubilize the resulting purple formazan crystals produced from metabolically viable cells. Absorbance was measured with an iMark micro-plate reader (Bio-Rad, USA) at 595 nm. Two wells were used for each concentration of the products assayed and three independent experiments were performed. Untreated and ethanol (1%) treated cells were used as controls while DOX (maximum tested concentration 69 *μ*M) was used as reference. The percentage of cytotoxic activity of the assayed compounds was determined by the following formula: cytotoxicity (%) = [1 − (optical density of treated cells − optical density DMSO)/(optical density of ethanol control cells − optical density DMSO)] × 100.

Medium inhibitory concentrations (IC_50_) represent the concentrations of the tested samples required to inhibit 50% cell proliferation and were calculated from the mean values of data from wells.

To confirm the effect of compound** 1** on the proliferation of CCRF-CEM and K562, cell suspensions (5 × 10^4^ cells/well) were treated in culture medium with different concentrations of** 1** (final volume of 200 *μ*L) and incubated in triplicate for 24, 48, and 72 h. Two independent experiments were performed. The number of viable cells at the different time points was counted by the trypan blue dye exclusion method using a hemocytometer (Neubaur, USA).

### 2.9. Cell Cycle Distribution by Flow Cytometry

CCRF-CEM and K562 cells placed in supplemented RPMI 1640 medium in 12-well plates (Greiner Bio-One, Germany) at a density of 5 × 10^5^/mL were treated with** 1** at a concentration equivalent to two times its IC_50_ for 72 h. Control cells were devoid of** 1** and negative controls contained 1% ethanol. After treatment, cells were harvested and washed twice with ice cold PBS. Then, cells were fixed with 70% cold ethanol and kept at 4°C for 24 h. Before analysis, the cells were washed twice with PBS and stained with a solution containing propidium iodide (PI, 50 *μ*g/mL, Sigma-Aldrich CO, USA) and RNase A (100 *μ*g/mL, Sigma-Aldrich CO, USA) in PBS (pH 7.4) for 30 min in the dark. DNA content was evaluated by flow cytometry. The relative distribution of at least 20,000 cells was analyzed using FlowJo software version 7.6.2 (Tree Star, Inc. OR, USA).

### 2.10. Assessment of Apoptosis by Annexin V/PI Double Staining Assay

CCRF-CEM and K562 cells (5 × 10^5^ cells/mL) placed in supplemented RPMI 1640 medium in 12-well plates (Greiner Bio-One, Germany) were treated for 72 h with compound** 1** at two times its IC_50_ value. After incubation, cells were harvested, washed, and suspended in cold PBS. The proportion of cells undergoing apoptosis was examined by double staining using allophycocyanin (APC) conjugated Annexin-V (BD Pharmingen, USA) and propidium iodide (Sigma-Aldrich CO, USA). Over the following hour, the fluorescence of ten thousand cells, including that of control groups, was assayed and populations were analyzed with FlowJo software version 7.6.2 (Tree Star, Inc., OR, USA). Percentages of Annexin-V positive cells (PI− or PI+) indicate early or late apoptosis, respectively.

### 2.11. Statistical Analysis

Data were analyzed by one-way analysis of variance (ANOVA) and means were compared using Bonferroni's comparison test, with *p* ≤ 0.05 considered as statistically significant. The inhibitory concentration (IC_50_) was calculated by log-Probit analysis responding to at least five concentrations at the 95% confidence level with upper and lower confidence limits.

## 3. Results and Discussion

**Figure 1:**
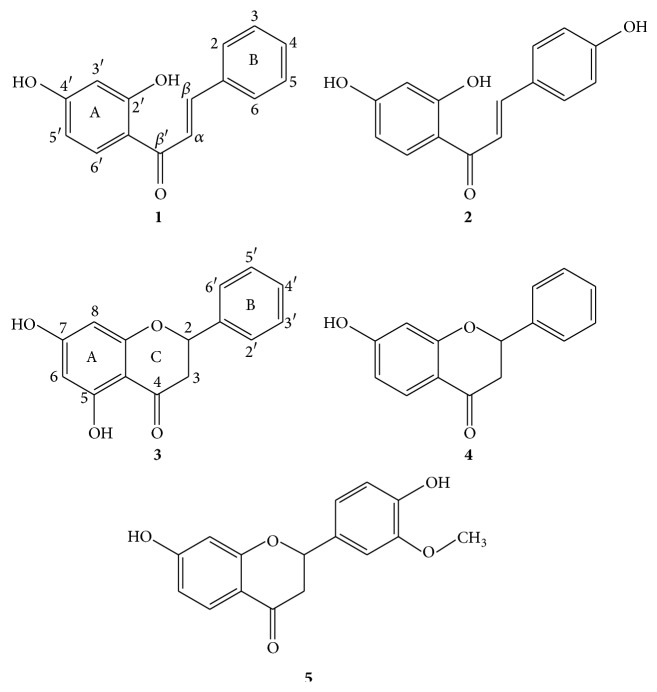
Chemical structures of 2′,4′-dihydroxychalcone (**1**), isoliquiritigenin (**2**), pinocembrin (**3**), 7-hydroxyflavanone (**4**), and 7,4′-dihydroxy-3′-methoxyflavanone (**5**).

In the current study, five compounds 2′,4′-dihydroxychalcone (**1**), isoliquiritigenin (**2**), pinocembrin (**3**), 7-hydroxyflavanone (**4**), and 7,4′-dihydroxy-3′-methoxyflavanone (**5**) (Figure 1) were obtained by bioguided fractionation from the ethanol extract of the aerial parts of* Flourensia oolepis*. This extract was selected among 51 extracts assayed by our group, after demonstrating an effective growth inhibition of a panel of pathogenic bacteria [7]. As far as we know, there was no information up to now about the metabolites responsible for the antibacterial activity of this extract. These results support the popular use of* F. oolepis* as an herb used for the treatment of respiratory tract infections such as bronchitis [23].

The presence of compounds** 1**,** 3,** and** 4** in* F. oolepis* has been previously reported [8, 24], but the presence of compounds** 2** and** 5** is reported here for the first time. It should be noted that while compounds** 1**–**4** have been frequently described in nature, this is not the case for compound** 5**. In fact, although there is considerable information available about the presence of its corresponding flavone (geraldone) [25, 26], there is very little describing the presence of** 5** in plants and there is a total absence of information concerning species native to Argentina.

As shown in Table 1, compound** 1** was the most effective, followed by** 3**. Both metabolites showed inhibitory effects against Gram-positive referents, with remarkable MIC values ranging from 15 to 62 *μ*g/mL. In relation to their bactericidal activity, it should be noted that the MBCs of** 1** and** 3** against* S. aureus* were similar to those obtained with the commercial antibiotics (31, 62, 10, and 60 *μ*g/mL, for** 1**,** 3**, gentamicin, and erythromycin, resp.).

As previously established in the literature, metabolites with MICs ≤ 100 *μ*g/mL are considered noteworthy [27, 28]. Based on this criterion, compound** 1** was clearly active against* E. faecalis*,* S. aureus,* and* P. mirabilis,* while** 3** was effective against both positive strains (Table 1). Since* S. aureus* is associated with respiratory diseases like bronchitis [29], the activity of compounds** 1** and** 3** against this pathogen supports the popular use of* F. oolepis* for the treatment of these affections [30]. 

On the other hand, the bacteriostatic activity of** 1** against the resistant strain of* P. mirabilis*, which was comparable to or even better than that of the commercial antibiotics used as referents, is highly encouraging. It should be pointed out that* P. mirabilis* is an important pathogen that has acquired resistance to clinically used antibiotics being the second most common cause of urinary tract infections [14].

The antibacterial activity of compounds** 1**–**4** has been previously described [31–34]; however, as far as we know, there are no reports of this inhibitory property for** 5**.

Certain structural features can be linked to the major antibacterial efficacy displayed by the chalcone** 1**. It has been reported that the carbonyl region is part of the active site of flavonoids [31], with the hydroxylations at positions 2′ and 4′ being important structural features for the antibacterial effect of chalcones [31, 35]. Interestingly, the other chalcone isolated, 2′,4′,4-trihydroxychalcone, commonly known as isoliquiritigenin (**2**), showed lower activity than its dihydroxylated counterpart (Table 1), demonstrating that the presence of a hydroxyl group at position 4 reduces effectiveness, a phenomenon that was also described by M. A. Alvarez et al. [35]. In addition,** 1** was more active for inhibiting bacterial growth than its respective flavanone** 4**.

With respect to flavanones, when comparing the activity of** 3** and** 4** against the Gram-positive strains, it was observed that the presence of a hydroxyl group at position 5 in compound** 3** may be responsible for its improved activity, since this substitution pattern constitutes the only structural difference with respect to** 4**. This agrees with a study of a number of structurally different flavonoids, which showed that hydroxylation at position 7 of flavanones is important for their antibacterial activity and the presence of an additional hydroxyl group at position 5 enhances the activity [31]. On the other hand, it was also reported that the presence of methoxy groups drastically reduces the antibacterial activity of flavonoids [31, 36]. This may be the reason why compound** 5** showed the weakest bacteriostatic activity among the flavanones.

The anticancer activity of compounds** 1**–**5** against a panel of two leukemia cell lines and their respective drug-resistant phenotypes was further evaluated by conducting MTT cell viability assays. As observed in Table 2, compound** 1** showed a strong cytotoxic effect against CCRF-CEM cells and their MDR counterpart CEM/ADR5000, as is evident from the IC_50_ values obtained, which were below 10 *μ*M, the value established by the US National Cancer Institute plant screening program for considering a pure compound as cytotoxic [37]. It is interesting to note that** 1** also showed a moderate inhibitory effect against K562 and Lucena 1 (Table 2) with IC_50_ values lower than 30 *μ*M. As observed, a lack of cross-resistance on P-gp-overexpressing cell lines was evidenced by the toxic effect exerted by** 1** over these (resistant index (RI) = IC_50_ resistant cells/IC_50_ sensitive cells = 1.5 and 1.1 against CEM/ADR5000 and Lucena 1 cells, resp.), in contrast to that found when cells were exposed to DOX (RI ≥ 518.8 and 16.8 against CEM/ADR5000 and Lucena 1, resp.). These results highlight the potential of** 1** as a therapeutic agent for the treatment of resistant phenotypes. Although its toxic effects toward different human and murine tumor cell types, such as MCF-7 (breast) [38], MGC-803 (gastric) [39], 26-L5 (colon), B16-BL6 (melanoma), A549 and LLC (lung), HeLa (cervix), HT-1080 (fibrosarcoma), HL 60 (promyelocytic leukemia), and PANC-1 (pancreatic) [40–42], have been described, this is the first report about the cytotoxicity of** 1** against ALL and CML cells and particularly against their MDR/P-gp sublines as a way to circumvent the reduced response of these resistant phenotypes to chemotherapy.

The toxic effect of** 1** was also determined against the noncancerous rapidly dividing cell line HEK293T [43] and against PBMC. From the IC_50_ values obtained, we saw that** 1** was highly selective against ALL cells, as evidenced by the selectivity indexes calculated (SI, ratio between IC_50_ value in nontarget cells to the IC_50_ value in tumor cells), with values greater than 6, taking into consideration previous classifications [44, 45]. When the medium growth inhibitory concentration of** 1** toward HEK293T (IC_50_ = 67.0 *μ*M) and PBMC (IC_50_ = 82.8 *μ*M) was compared to that of CCRF-CEM (IC_50_ = 6.6 *μ*M), SIs of 10.1 and 12.5 were, respectively, observed, while in the case of CEM/ADR5000 (IC_50_ 9.9 *μ*M), the IC_50_ ratios were 6.8 and 8.4, respectively. On the other hand, compounds with SIs between 3 and 6 were rated as moderately selective [44, 45]. According to the ratios between the IC_50_ obtained for PBMC and those obtained for K562 cells (IC_50_ = 27.5 *μ*M) and for Lucena 1 (IC_50_ = 30.0 *μ*M) cells, compound** 1** meets this criterion (SI = 3.0 and 2.8, resp.). It is worth noting that** 1** also showed a low toxic effect when assayed against normal hepatocytes (IC_50_ 34 *μ*g/mL, by the trypan blue exclusion method) [46].

The selective activity of** 5** is also interesting, showing moderate toxicity against CCRF-CEM and CEM/ADR5000 in contrast to its complete lack of effectiveness (IC_50_ > 100 *μ*M) on the rest of the assayed cells.

Comparing the cytotoxic activity of chalcones** 1** and** 2**, the presence of an additional hydroxyl group at position 4 of compound** 2** would not prove favorable for its toxicity, as reflected by its higher IC_50_ values (51.9–>100 *μ*M) with respect to** 1** (6.6–30.0 *μ*M). This is in complete agreement with Li et al. [40], who tested these compounds against a panel of different tumor cell lines and reported that the toxicity exerted by** 1** broadly exceeded that of compound** 2**. From our results, we can infer that the presence of 4-OH negatively influences both cytotoxic and antibacterial activity.

The strong cytotoxic activity exhibited by** 1** in comparison to the low potency exerted by** 4** matches that reported by Pouget et al. [38], who found that** 1** was more effective at inhibiting MCF-7 cell proliferation than its corresponding flavanone** 4**. The presence of a hydroxyl group at position C-5 made no difference to the activity of compounds** 3** and** 4**, in contrast to that observed with respect to the antibacterial effect exerted by these compounds. The substitution pattern of the B-ring in flavanone** 5** with respect to** 4** increased the compound's toxicity against CCRF-CEM and its MDR counterpart (see Table 2).

**Figure 2:**
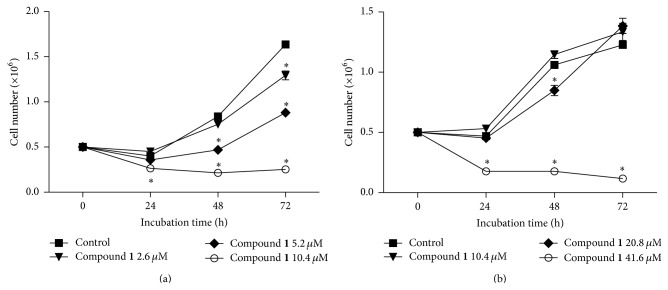
Time- and dose-dependent effects of compound** 1** on CCRF-CEM (a) and K562 (b) cells. Cell viability was quantified by using the trypan blue dye exclusion method. The results are expressed as mean ± SEM. Significant differences were labeled with asterisks (*p* < 0.05) compared to 1% ethanol control group at each time (ANOVA followed by Bonferroni's test).

When the number of viable cells was determined at different time points by a trypan blue exclusion test, it was observed that** 1** exerted a negative effect on normal cell proliferation of both CCRF-CEM (Figure 2(a)) and K562 (Figure 2(b)) cell lines in a time- and dose-dependent manner. As shown in Figure 2(a), compound** 1** at 2.6 *μ*M showed a significant difference (*p* < 0.05) in the number of CCRF-CEM viable cells with respect to the ethanol control at 72 h from the beginning, while this effect was already observed at 48 h when 5.2 *μ*M of** 1** was added. When K562 cells were treated with** 1** at 20.8 *μ*M for 48 h, a significant decrease (*p* < 0.05) was observed in the number of viable cells compared to the ethanol control and, when the concentration of** 1** was increased to 41.6 *μ*M, this difference (*p* < 0.05) was already observed at 24 h (Figure 2(b)).

**Figure 3:**
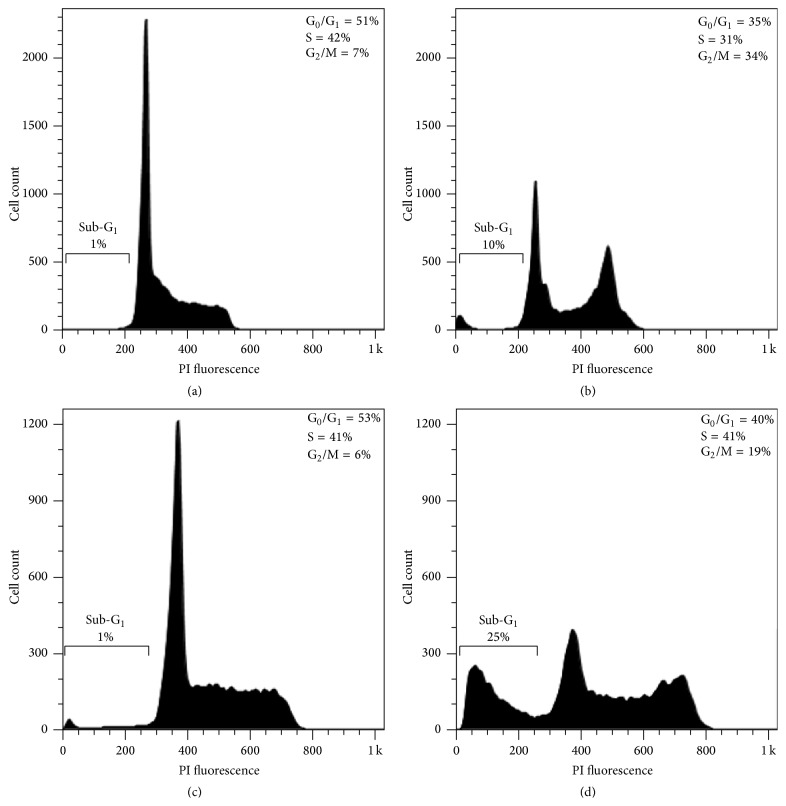
CCRF-CEM and K562 cells treated with 1% ethanol (controls (a) and (c), resp.) or with** 1** at 2 × IC_50_ ((b) and (d), resp.) for 72 h were stained with PI for cell cycle phase distribution analysis by flow cytometry. The percentages of cells in each phase, including sub-G1 (hypodiploid), are indicated.

When exposed to cytotoxic agents, cells may suffer death or a stop in their cell cycle, which in turn can trigger death by apoptosis if cells cannot overcome the damage [47]. Apoptosis is a common mechanism involved in the toxicity of many anticancer drugs, including those from natural origin [48]. Hence, due to the strong compound** 1**-induced cytotoxicity and since the mechanism underlying this effect in the leukemic cells, CCRF-CEM, and K562 cells was uncertain, we performed flow cytometric experiments to dissect the possible mode of action beyond the toxic effect of** 1**. As depicted in Figure 3(b), analysis of the DNA contents of CCRF-CEM cells indicated that treatment with** 1** at 2 × IC_50_ for 72 h produced a cell cycle arrest of 34% of the cells in the G_2_/M phase compared to 7% of cells in this phase in the ethanol control (Figure 3(a)). K562 cells also showed an increase in the proportion of cells in the G_2_/M phase (19%) (Figure 3(d)) compared to the ethanol control (6%) (Figure 3(c)). Given the key role that the G_2_/M checkpoint plays in the maintenance of chromosomal integrity, allowing cells to repair DNA damage before entering mitosis [49], blockage of the cell cycle at this stage by compound** 1** emerges as a potential pharmacological strategy for the treatment of leukemia. The arrest observed was also accompanied by an increase in the hypodiploid sub-G1 population from 1% in the ethanol controls (Figures 3(a) and 3(c)) to 10% and 25% for CCRF-CEM and K562 cells, respectively (Figures 3(b) and 3(d)), indicating the presence of cell death.

**Figure 4:**
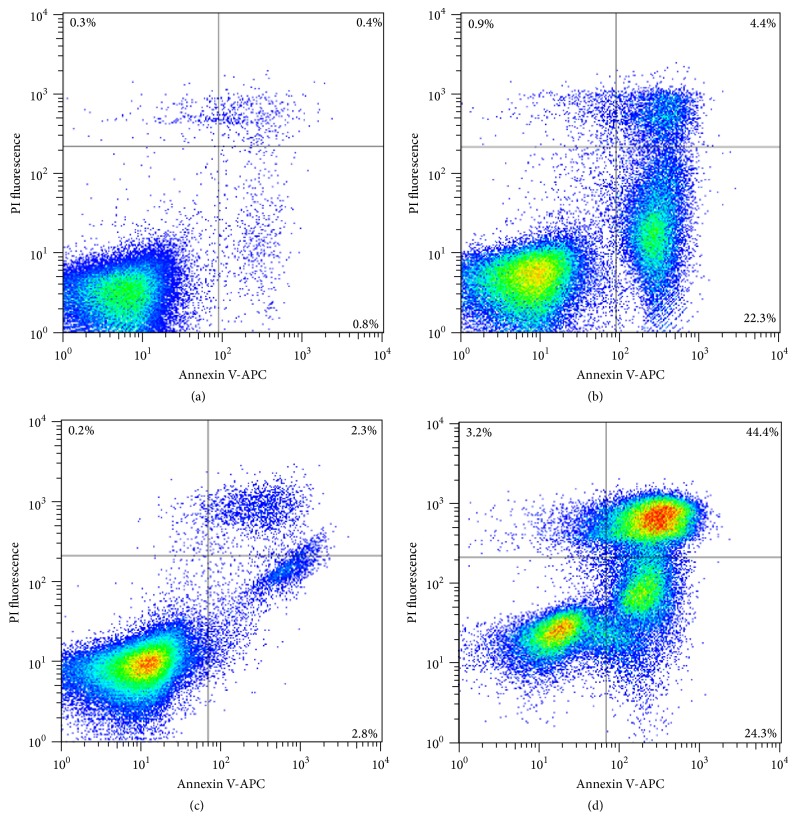
Effects of compound** 1** on cell death process. CCRF-CEM and K562 cells were incubated for 72 h with 1% ethanol (controls (a) and (c), resp.) or with 2 × IC_50_ of** 1** ((b) and (d), resp.). Apoptotic effect of** 1** was evaluated by flow cytometry analysis after double staining with Annexin V-APC and PI. Annexin V-APC intensity is represented on the *x*-axis and PI fluorescence intensity is represented on the *y*-axis.

To further determine the induction of apoptosis, the flipping of phosphatidylserine to the outer membrane as a hallmark of apoptosis was analyzed using Annexin V staining. As shown in Figures 4(b) and 4(d), compound** 1** at 2 × IC_50_ induced apoptosis in CCRF-CEM and in K562 at 72 h of treatment, reaching 26.7% and 68.7%, respectively, of early and late apoptotic cells.

In agreement with our results, Lou et al. [50, 51] reported that the exposure of the human gastric cancer cell line MGC-803 to** 1** resulted in a dose-dependent G_2_/M phase cell cycle arrest, accompanied by apoptosis, but these effects have not been described yet for ALL and CML cells.

## 4. Conclusions

This work extends the currently available information about the chemical composition and activity of* F. oolepis *and offers a set of bioactive molecules that can be used as a model for the design of improved antibacterial and cytotoxic compounds. The antibacterial activity observed for compound** 1**, in addition to its strong and selective cytotoxicity, highlights its potential as a lead compound to fight against pathogenic bacteria and ALL and CML cells, including their MDR phenotypes.

## Supplementary Material

Supplementary Material provides the spectroscopic data of the isolated compounds (^1^H and ^13^C NMR spectra).

## Figures and Tables

**Table 1:** Antibacterial activity of compounds **1**–**5** isolated from *Flourensia oolepis* extract.

Compound	MIC (MBC) (*μ*g/mL)				
	*E*. *coli*	*P*. *aeruginosa*	*P*. *mirabilis*	*E*. *faecalis*	*S*. *aureus*
2′,4′-Dihydroxychalcone (1)	250 (500)	250 (>500)	62 (125)	62 (125)	31 (31)

Isoliquiritigenin (2)	>500 (>500)	>500 (>500)	>500 (>500)	500 (>500)	250 (500)

Pinocembrin (3)	500 (500)	>500 (>500)	>500 (>500)	62 (125)	15 (62)

7-Hydroxyflavanone (4)	500 (500)	500 (>500)	>500 (>500)	250 (500)	250 (250)

7,4′-Dihydroxy-3′-methoxyflavanone (5)	>500 (>500)	>500 (>500)	>500 (>500)	500 (>500)	250 (>500)

Gentamicin	4 (8)	4 (4)	10 (10)	8 (10)	8 (10)

Erythromycin	125 (>500)	62 (500)	500 (>500)	1 (30)	1 (60)

**Table 2:** Anticancer effects of compounds **1**–**5** isolated from *Flourensia oolepis* extract toward sensitive and resistant leukemic cells.

Compounds	IC_50_ (*μ*M) values and 95% confidence limits (lower, upper)			
	CCRF-CEM	CEM/ADR5000	K562	Lucena 1
2′,4′-Dihydroxychalcone (1)	6.6 (3.3–14.1)	9.9 (5.8–18.3)	27.5 (14.6–52.0)	30.0 (14.6–64.9)
Isoliquiritigenin (2)	58.1 (22.6–148.7)	51.9 (26.9–99.1)	>100	>100
Pinocembrin (3)	58.9 (16.4–210.3)	53.5 (19.5–145.9)	>100	>100
7-Hydroxyflavanone (4)	72.8 (30.0–175.2)	66.2 (27.5–160.7)	>100	>100
7,4′-Dihydroxy-3′-methoxyflavanone (5)	24.8 (10.8–57.3)	30.4 (15.4–60.1)	>100	>100
Doxorubicin	0.133 (0.0–0.3)	>69	4.1 (1.6–11.4)	>69
