# Supplementary material for: Antibacterial and Cytotoxic Activity of Compounds Isolated from Flourensia oolepis
Source: Evid Based Complement Alternat Med. 2015 Dec 27;2015:912484. doi: 10.1155/2015/912484 (PMC4706877; doi:10.1155/2015/912484)
Supplement: Supplementary file 1 — Supplementary Material provides the spectroscopic data of the isolated compounds (1H and 13C NMR spectra). [file 912484.f1.pdf]

Antibacterial and cytotoxic activity of compounds isolated from *Flourensia oolepis*

Mariana Belén Joray,[1] Lucas Daniel Trucco,[2] María Laura González,[1] Georgina Natalia Díaz Napal,[1] Sara María Palacios,[1] José Luis Bocco,[2] and María Cecilia Carpinella[1*]

[1] *Fine Chemicals and Natural Products Laboratory, School of Chemistry, Catholic University of Córdoba, Avda Armada Argentina 3555, X5016DHK Córdoba, Argentina*

[2] *CIBICI CONICET and Department of Clinical Biochemistry, Faculty of Chemical Science, National University of Córdoba, Haya de la Torre and Medina Allende, Córdoba, Argentina*

Supplementary Material

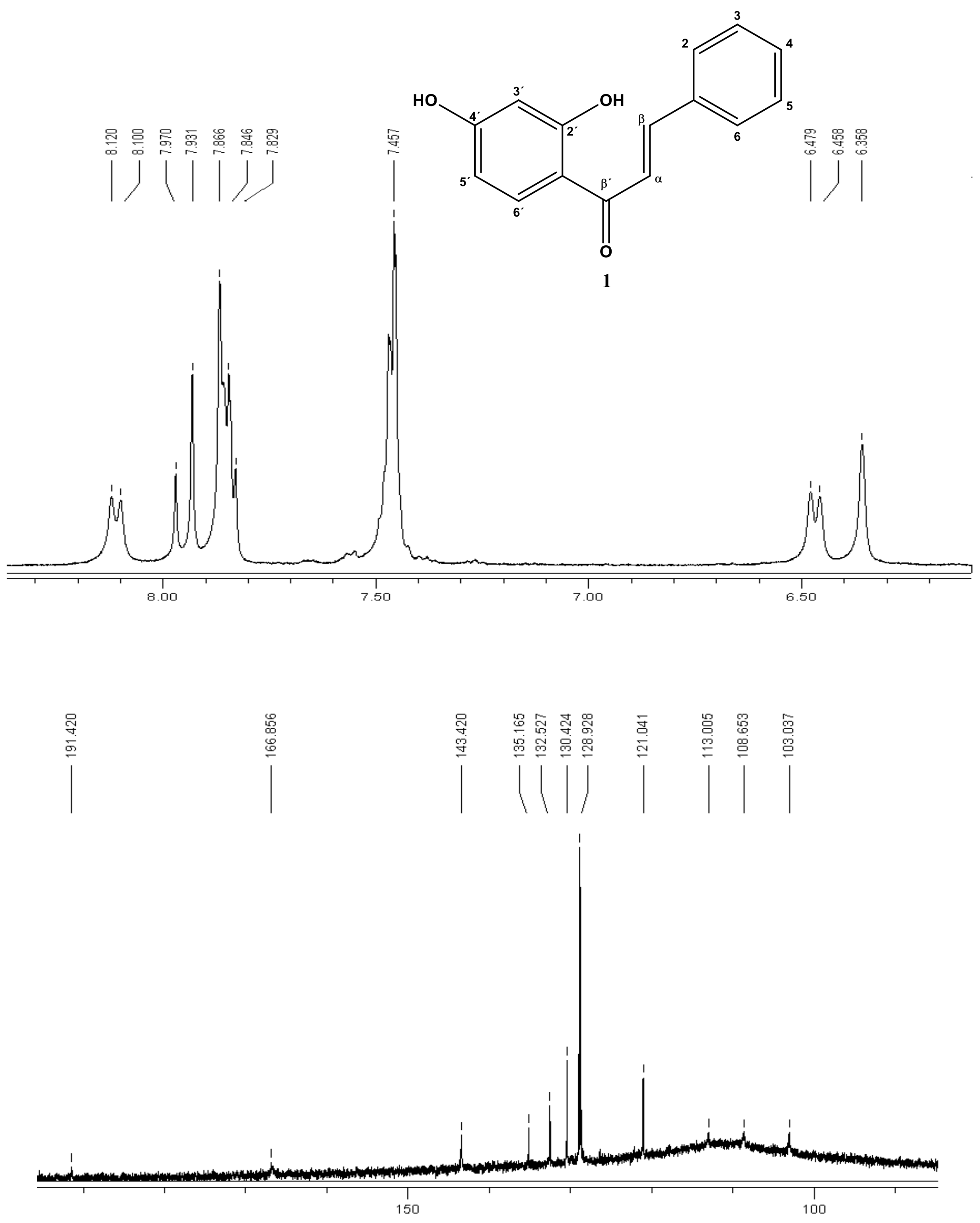

Figure 1. NMR spectra of 2´,4´-dihydroxychalcone (**1**). ^{1}H NMR (400 MHz, CD6O): δ (ppm) 8.11 (1 H, d, *J*=8.0 Hz ,H-6´), 7.95 (1 H, d, *J*=15.6 Hz ,H-α), 7.85 (1 H, d, *J*=14.8 Hz ,H-β), 7.85-7.45 (5 H, m , H-2-6), 6.47 (1 H, d, *J*=8.4 Hz ,H-5´), 6.35 (1 H, s , H-3´).^{13}C NMR (50 MHz, CD6O)**:** δ (ppm) 103.0 (C-3´); 108.6 (C-5´); 113.0 (C-1´); 121.0 (C-α); 128.9 (C-2,3,5,6); 130.4 (C-4); 132.5 (C-6´); 135.2 (C-1); 143.4 (C-β); 166.8 (C-2´,4´); 191.4 (C=O).

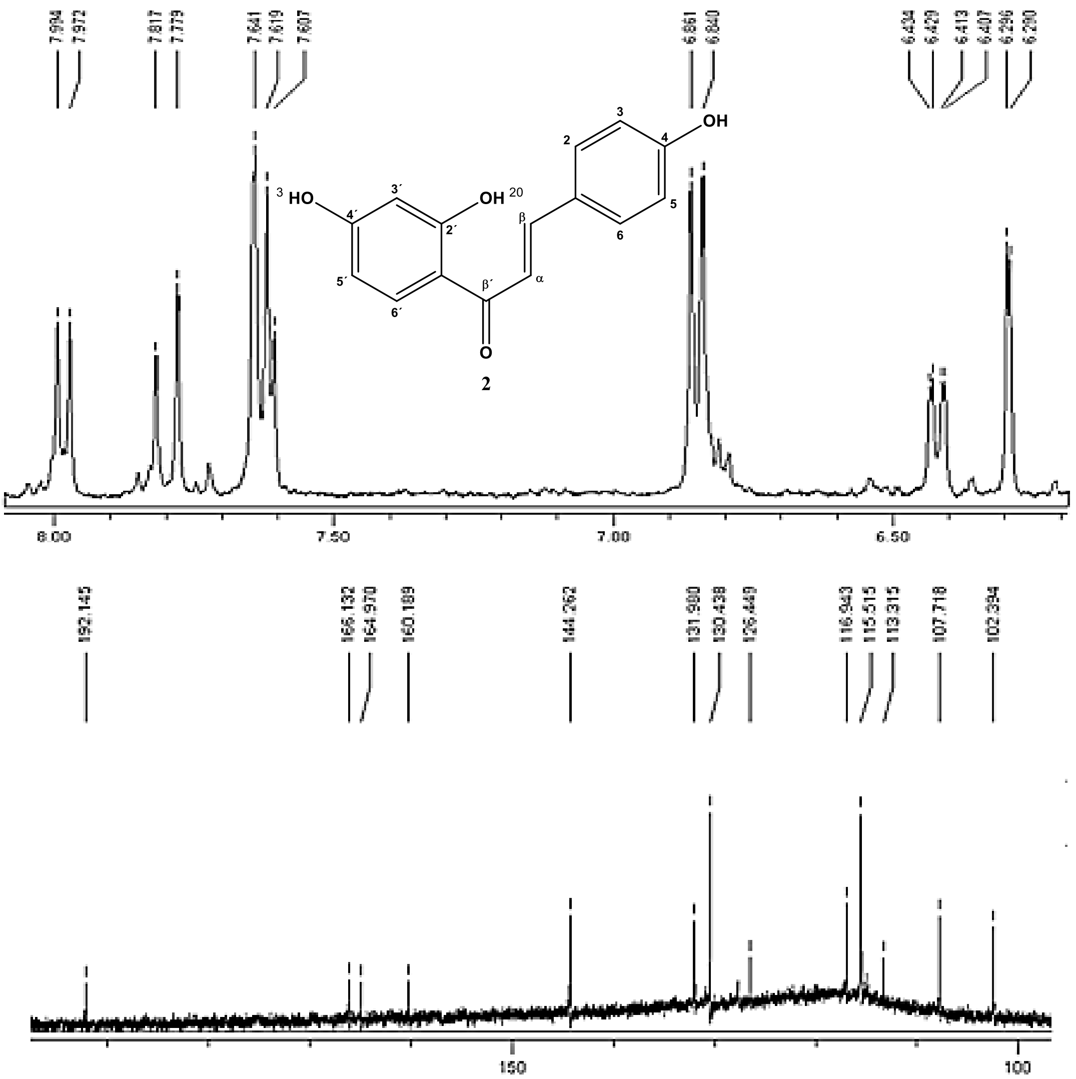

Figure 2. NMR spectra of isoliquiritigenin (**2**). ^{1}H NMR (400 MHz, CD6O): δ (ppm) 7.96 (1 H, d, *J* = 8.8 Hz, H-2´), 7.79 (1 H, d, *J* = 15.2 Hz, H-β), 7.63 (2 H, d, *J* = 9.2 Hz, H-2, H-6,), 7.62 (1 H, d, *J* = 14.8 Hz, H-α), 6.85 (2 H, d, *J* = 8.8 Hz, H-3, H-5), 6.42 (1 H, dd, *J* = 8.8, 2.4 Hz, H-3´), 6.29 (1 H, d, *J* = 2.4 Hz, H-5´). ^{13}C NMR (50 MHz, CD6O): δ (ppm) 102.4 (C-5´), 107.7 (C-3´), 113.3 (C-1´), 115.2 (C-3, 5), 116.9 (C-α), 126.4 (C-1), 130.4 (C-2, 6), 131.9 (C-2´), 144.3 (C-β), 160.2 (C-4), 164.9 (C-4´), 166.1 (C-6´), 192.1 (C=O).

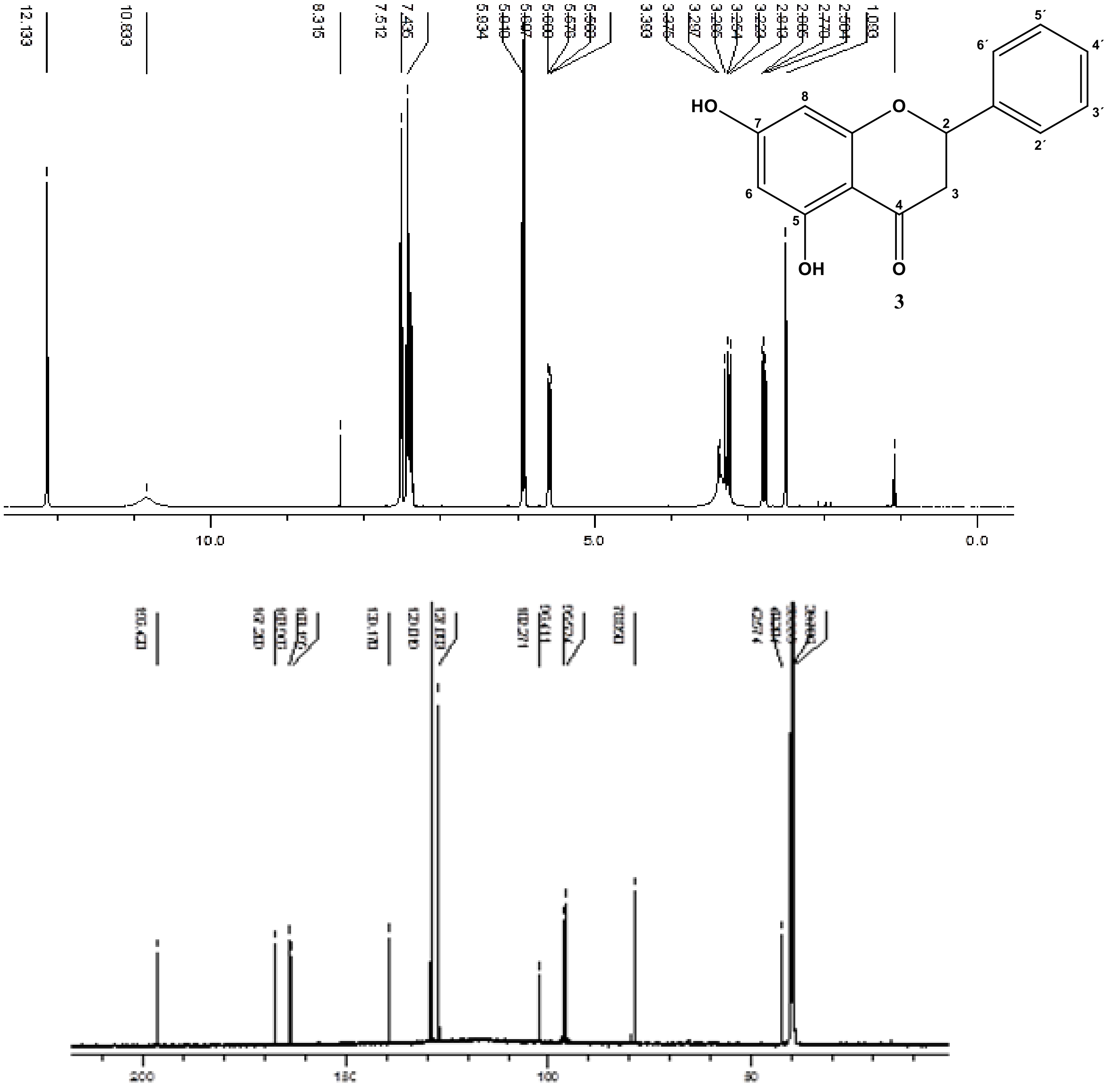

Figure 3. NMR spectra of pinocembrin (**3**). ^{1}H NMR (400 MHz, DMSO-d_6): δ (ppm) 7.41 (5H, m, H-20-60), 6.01 (1H, d, *J* = 2.2 Hz, H-8), 5.52 (1H, d, *J* = 2.2 Hz, H-6), 5.44 (1H, dd, *J* = 12.8, 3.2 Hz, H-2), 3.06 (1H, dd, *J* = 12.8, 17.2 Hz, H-3ax), 2.77 (1H, dd, *J* = 17.2, 3.2 Hz, H-3eq). ^{13}C NMR (50 MHz, DMSO-d_6): δ (ppm) 40.4 (C-3), 80.2 (C-2), 95.9 (C-8), 96.8 (C-6), 102.7 (C-10), 127.5 (C-20/60), 129.4 (C-40) 129.5 (C-30/50), 139.6 (C-10), 163.6 (C-9), 164.4 (C-5), 167.6 (C-7), 196.7 (C-4).

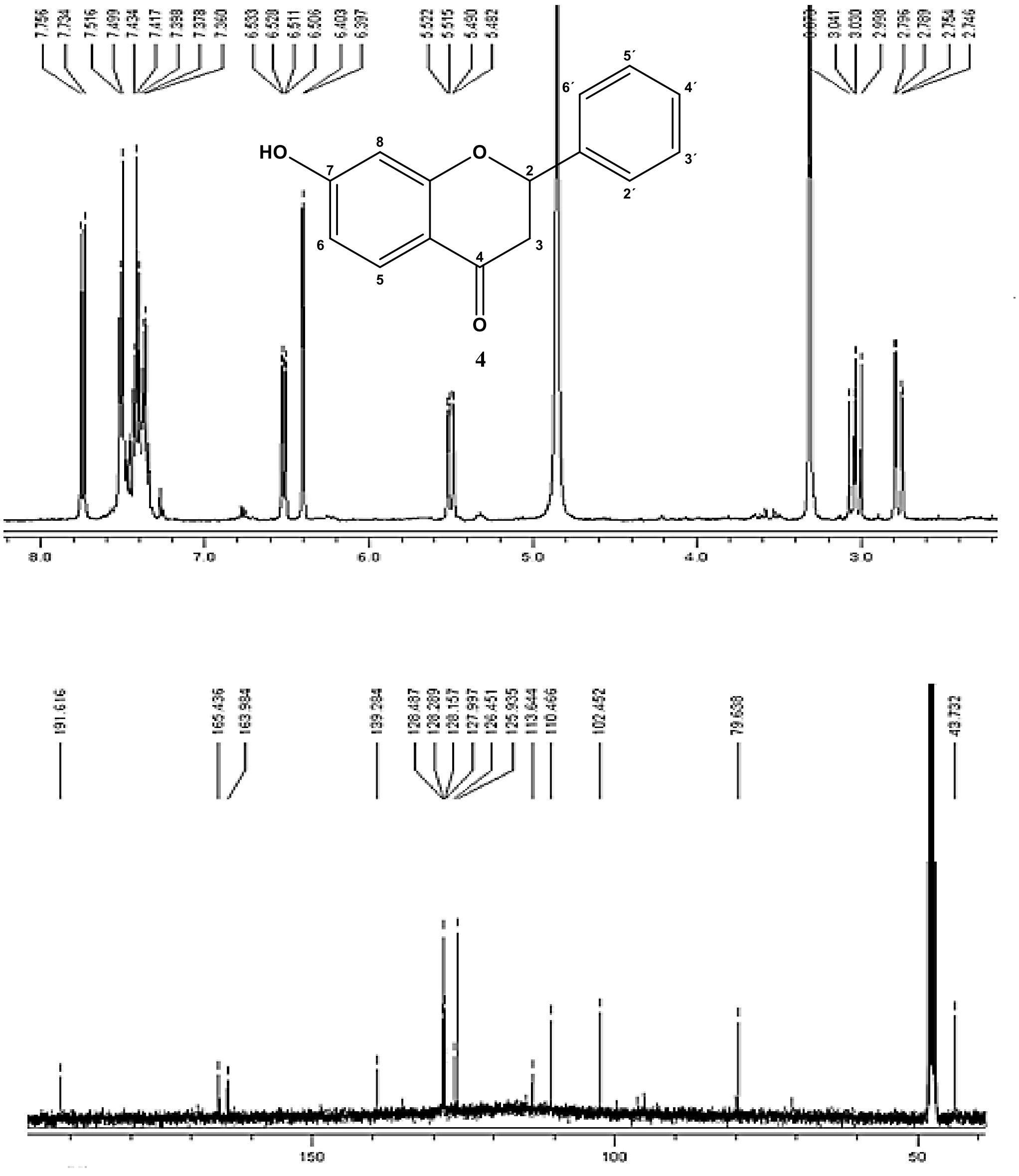

Figure 4. NMR spectra of 7-hydroxyflavanone (**4**). ^{1}H NMR (400 MHz, CD6O)**:** δ (ppm) 7.74 (1 H, d, *J* = 8.8 Hz, H-5), 7.50 (2 H, d, *J* = 6.8 Hz, H-2´, H-6´), 7.42 (2 H, t, *J* = 7.2 Hz, H-3´, H-5´), 7.36 (1 H, t, *J* = 7.2 Hz, H-4´), 6.52 (1 H, dd, *J* = 8.8, 2.0 Hz, H-6), 6.40 (1 H, d, *J* = 2.4 Hz, H-8), 5.50 (1 H, dd, *J* = 12.8, 2.8 Hz, H-2), 3.03 (1 H, dd, *J* = 17.2, 12.8 Hz, H-3ax), 2.79 (1 H, dd, *J* = 16.8, 3.0 Hz, H-3eq). ^{13}C NMR (50 MHz, CD6O): δ (ppm) 43.7 (C-3), 79.6 (C-2), 102.4 (C-8), 110.5 (C-6), 113.4 (C-10), 125.9 (C-2´), 126.4 (C-6´), 128 (C-4´), 128.2 (C-3´), 128.3 (C-5´), 128.4 (C-5), 139.3 (C-1´), 163.3 (C-7), 165.4 (C-9), 191.6 (C=O).

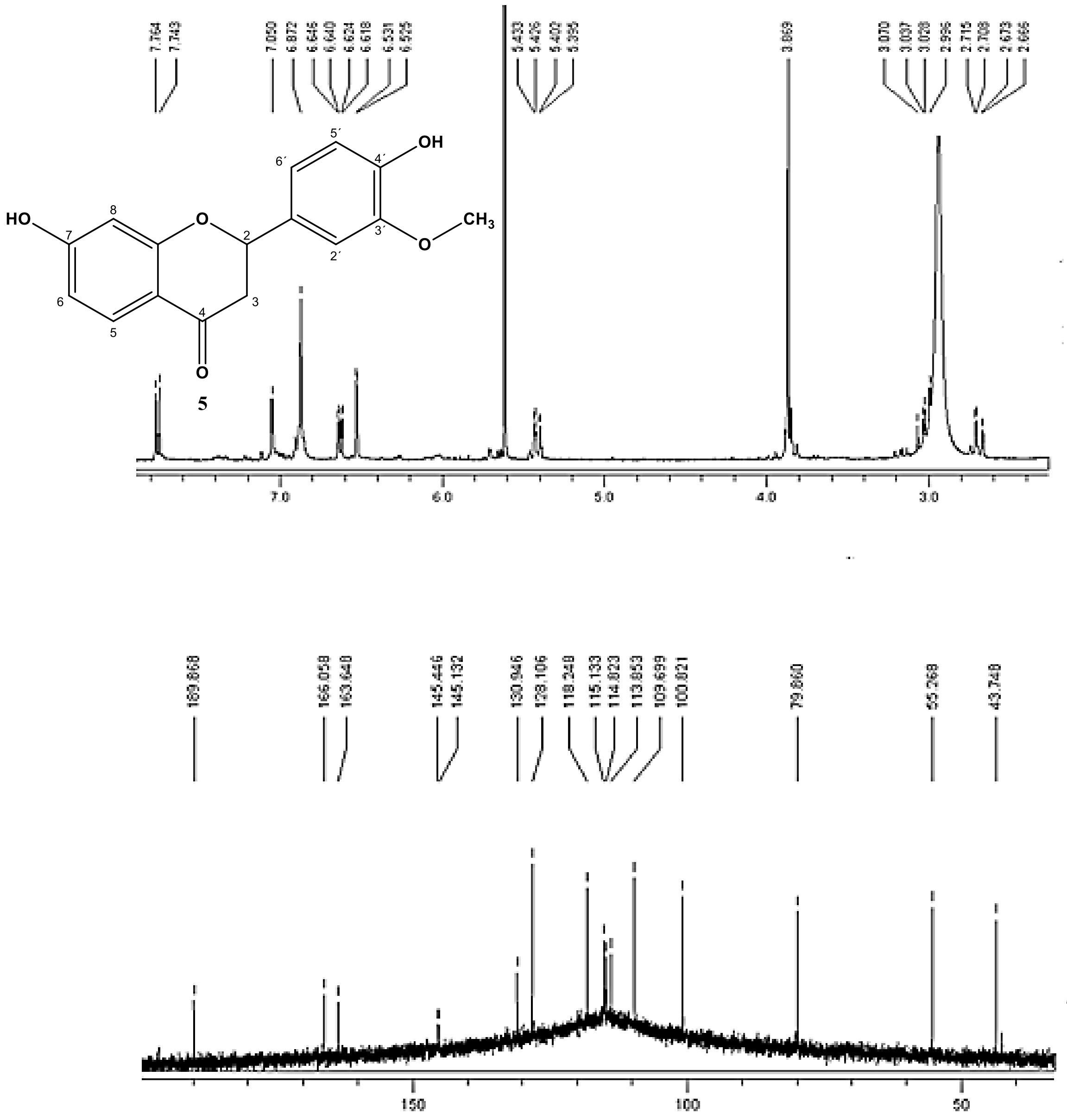

Figure 5. NMR spectra of 7,4´-dihydroxy-3´-methoxyflavanone (**5**). ^{1}H NMR (400 MHz, CD6O): δ (ppm) 7.75 (1 H, d, *J* = 8.4 Hz, H-5), 7.05 (1 H, s, H-2´), 6.88 (1 H, d, *J* = 7.6 Hz, H-5´), 6.88 (1 H, dd, *J* = 12.8, 5.2 Hz, H-6´), 6.64 (1 H, dd, *J* = 9.2, 2.8 Hz, H-6), 6.53 (1 H, d, *J* = 2.4 Hz, H-8), 5.41 (1 H, dd, *J* = 12.4, 2.8 Hz, H-2), 3.87 (3 H, s, O-CH_3), 3.03 (1 H, dd, *J* = 16.6, 12.8 Hz, H-3ax), 2.69 (1 H, dd, *J* = 16.8, 2.8 Hz, H-3eq). ^{13}C NMR (50 MHz, CD6O): δ (ppm) 43.8 (C-3), 55.3 (O-CH_3), 79.8 (C-2), 100.8 (C-8), 109.7 (C-6), 113.8 (C-2´),114.8 (C-10), 115.1 (C-5´), 118.2 (C-6´), 128.1 (C-5), 130.9 (C-1´), 145.1 (C-4´), 145.4 (C-3´), 163.6 (C-9), 166.0 (C-7), 189.9 (C=O).
